# Head and neck contrast-enhanced CT for identification of internal carotid artery stenosis progression on the affected side after treatment for oral squamous cell carcinoma

**DOI:** 10.1007/s11282-012-0099-8

**Published:** 2012-08-11

**Authors:** Hiroaki Ikawa, Kazumichi Sato, Morio Tonogi, Gen-yuki Yamane, Masako Kimura, Satoshi Tatsuno, Yutaka Aoyagi, Akira Katakura

**Affiliations:** 1 Department of Oral Medicine, Oral and Maxillofacial Surgery, Tokyo Dental College, 5-11-13 Sugano, Ichikawa, Chiba 272-8513 Japan; 2 Oral Cancer Center, Tokyo Dental College, 5-11-13 Sugano, Ichikawa, Chiba 272-8513 Japan; 3 Department of Oral and Maxillofacial Surgery, Nihon University School of Dentistry, 1-8-13 Kanda-Surugadai, Chiyoda-ku, Tokyo, 101-8310 Japan; 4 Department of Radiology, Tokyo Dental College, Ichikawa General Hospital, 5-11-13 Sugano, Ichikawa, Chiba 272-8513 Japan

**Keywords:** Radiological diagnosis, Retrospective study, Radiation therapy, Plaque, NASCET

## Abstract

**Objectives:**

To determine the incidence of progressive internal carotid artery (ICA) stenosis by head and neck contrast-enhanced computed tomography (CT) in 82 patients who underwent surgery, chemotherapy, or combination therapy for oral squamous cell carcinoma (OSCC).

**Methods:**

The study included 82 patients who underwent head and neck contrast-enhanced CT after surgery alone or combined surgery and chemotherapy for OSCC at the Department of Oral and Maxillofacial Surgery of Ichikawa General Hospital, Tokyo Dental College, or Tokyo Dental College Oral Cancer Center between December 2002 and March 2010.

**Results:**

Comparison with previously obtained head and neck contrast-enhanced CT images revealed progressive arterial stenosis of the ICA in five patients with a mean age of 62.0 years. All five patients were male, and their OSCC sites were the tongue in two, the floor of the mouth in two, and the mandibular gingiva in one. Tumor resection and neck dissection were performed for four patients and tumor resection alone for one patient. Four patients underwent chemotherapy. ICA stenosis occurred on the same side as the tumor in all five patients.

**Conclusions:**

The results of this study suggest that, given the possibility of post-treatment vascular events, attention must be paid to subsequent changes in the ICA over time. The results also indicate the usefulness of head and neck contrast-enhanced CT in identifying such problems.

## Introduction

Head and neck contrast-enhanced computed tomography (CT) is a valuable imaging technique for determining tumor location and cervical lymph node status in patients with oral squamous cell carcinoma (OSCC). It can also be used to evaluate cervical lymph node status during post-treatment follow-up, making head and neck contrast-enhanced CT beneficial for OSCC patients.

Recently, several studies have reported an increased risk of cerebral infarction owing to carotid wall thickening in patients who have undergone radiotherapy for head and neck cancers [1–4], and other reports have described the occurrence of a cerebral infarction during the perioperative period among patients undergoing surgical treatment [5–8]. However, as far as we are aware, no studies have investigated the usefulness of head and neck contrast-enhanced CT for identifying progressive stenosis of the internal carotid artery (ICA) after surgery, chemotherapy, or combination therapy.

The objective of this study was to determine the incidence of progressive ICA stenosis in 82 patients who underwent surgery, chemotherapy, or combination therapy for OSCC by follow-up head and neck contrast-enhanced CT.

## Patients and methods

A total of 82 patients were included in the study. All the patients underwent head and neck contrast-enhanced CT after surgery alone or combined surgery and chemotherapy for OSCC at the Department of Oral and Maxillofacial Surgery of Ichikawa General Hospital, Tokyo Dental College, or Tokyo Dental College Oral Cancer Center over an approximately 7-year period spanning December 25, 2002 to March 31, 2010. These examinations were performed for the purpose of evaluating the primary cancer location and the cervical lymph nodes. None of the examinations was performed for suspected carotid occlusive disease.

All the patients underwent clinically indicated neck CT with a helical multidetector array CT scanner after bolus intravenous administration of non-ionic contrast material. The iodinated contrast material (Iopamiron^®^ 300; Bayer Schering Pharma, Leverkusen, Germany) was administered in the following manner: 100 mL was injected via a CT injector after obtaining a investigative topogram. All of the patients were evaluated by post-contrast neck CT images, which were obtained by use of a multidetector array system (Brilliance CT 64-channel scanner or Mx8000 IDT 16 CT; Philips, Amsterdam, The Netherlands). Scanning was performed at 120 kVp with a maximum tube current of 500 mA (adjustable effective mA depending on the patient body habitus).

Because the objective of the study was to determine the usefulness of this imaging modality for identifying worsening stenosis of the ICA over time, imaging was performed during post-treatment follow-up every 3–6 months, and all images were evaluated by the same practitioner.

**Fig. 1:**
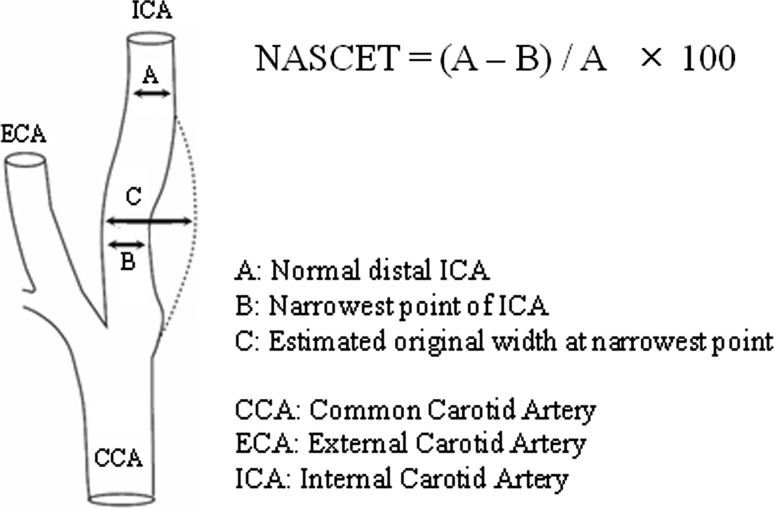
For each projection, three measurements are made to record the luminal diameter of the site of greatest narrowing (measure* B*) and the artery beyond the plaque and bulb (measure* A*). Using the worst projection, with measure* A* minus measure* B* as the numerator and measure* A* as the denominator, the percentage stenosis is calculated

The items evaluated included the OSCC site, TNM classification, treatment method, hypertension, diabetes mellitus, hyperlipidemia, body mass index (BMI), smoking habit, drinking habit, stenosis site, and stenosis degree. In this study, the diagnostic CT criteria used for carotid artery stenosis were the North American Symptomatic Carotid Endarterectomy Trial (NASCET) stenosis criteria [9]. The NASCET method, a standard measurement comparison for stenosis, was defined as the ratio between the lumen diameter at the stenosis and the normal lumen diameter at a distal region with no stenosis, and the percentage stenosis is calculated (Fig. 1).

## Results

The results are shown in Table 1. Progressive stenosis of the ICA was identified in five of the 82 patients, who were all male and had a mean age of 62.0 years (range 53–76 years). In these five patients, the OSCC sites were the tongue for two, the floor of the mouth for two, and the mandibular gingiva for one. The treatments involved tumor resection and neck dissection for four patients and tumor resection alone for one patient. Four patients underwent chemotherapy. For all five patients, the progressive deterioration of ICA stenosis was present on the same side as the tumor. None of the patients had an exceptionally high BMI, but all the patients had a history of smoking and drinking. Blood tests for all patients revealed hyperlipidemia (Table 2). In Cases 2 and 5, in particular, treatment was required for the ICA stenosis (Table 3). Case 2, in which surgical treatment was required for ICA stenosis, is a typical case.

**Table 1:** Summary of 5 OSCC cases with ICA stenosis

	Sex	Age	OSCC location	TNM classification	Surgical therapy	Chemotherapy	Site of stenosis
Case 1	M	53	Mouth floor (left side)	T2 N1 M0	Tumor excision Left side SOHNO	+	ICA origin stenosis (both sides)
Case 2	M	64	Tongue (right ride)	T2 N0 M0	Tumor excision Right side RND	**+**	ICA origin stenosis (right side)
Case 3	M	64	Mouth floor (left side)	T2 N2c M0	Tumor excision Left side RND	+	ICA origin stenosis (left side)
Case 4	M	76	Mandibular gingiva (right side)	T1 N0 M0	Tumor excision Right side RND	**+**	ICA origin stenosis (right side)
Case 5	M	53	Tongue (right side)	T2 N0 M0	Tumor excision	**−**	ICA origin stenosis (right side)

*OSCC* oral squamous cell carcinoma,* ICA* internal carotid artery,* RND* radical neck dissection

**Table 2:** Summary of findings on risk factors for ICA stenosis

	Hypertension	Diabetes mellitus	Hyperlipidemia	BMI	Smoking habits	Drinking habits
Case 1	−	−	+	17.2	+	**+**
Case 2	−	+	+	24.2	**+**	+
Case 3	+	−	+	25 1	+	+
Case 4	+	−	+	24.4	+	+
Case 5	+	+	**+**	18.8	**+**	+
Reference range	SBP >l60 mmHg DBP >95 mmHg	FBS >126 mg/dL	Tcho >200 mg/dL	22.0 kg/m^2^	+ or −	+ or −

*SBP* systolic blood pressure,* DBP* diastolic blood pressure,* FBS* fasting blood sugar,* Tcho* total cholesterol,* BMI* body mass index

**Table 3:** Summary of findings on ICA stenosis and outline

	Side of ICA origin stenosis		Preoperative ICA evaluation of OSCC (NASCET)	Started period of stenosis progression from each therapy	Post-operative worst ICA stenosis rate of OSCC (NASCET)	Follow up period (years)	Therapeutics
Case 1	Both sides		Right side: 20 % Left side: 0 %	9 years and 5 months	Right side: 70 % Left side: 35 %	10	Follow-up
Case 2	Right side		Right side: 42 %	10 years and 7 months	Right side: 70 %	12	Surgical therapy: carotid artery stenting
Case 3	Left side		Left side: 67 %	1 month	Left side: 80 %	2	Follow-up
Case 4	Right side		Right side: 30 %	5 months	Right side: 40 %	4	Follow-up
Case 5	Right side		Right side: 0 %	6 months	Right side: 50 %	3	Drug therapy: cilostazol, aspirin, telmisartan

*OSCC* oral squamous cell carcinoma,* NASCET* North American Symptomatic Carotid Endarterectomy Trial,* ICA* internal carotid artery

**Fig. 2:**
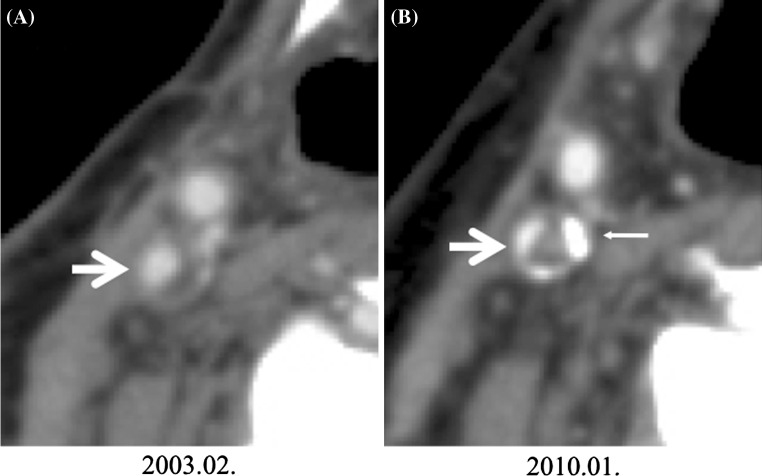
Case 2. **a** ICA stenosis shown by the NASCET ratio of 42 % (*arrow*). **b** Progression of ICA stenosis indicated by the NASCET ratio of 70 % (*arrow*). The *thin arrow* shows calcified plaque of the ICA

**Fig. 3:**
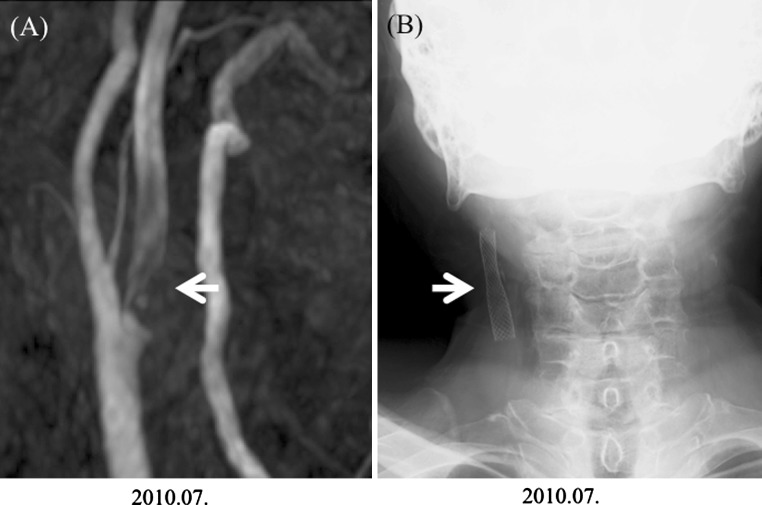
Case 2. **a** Magnetic resonance angiograph before carotid artery stenting (*arrow*). The low signal area reveals plaque from the origin of the ICA to the cranial side, which has been narrowed by the plaque that is present. **b** Post-operative carotid artery stenting (*arrow*)

Case 2 was a 64-year-old man with a tumor on the right side of the tongue that was resected in December 1997 (T2N0M0 Stage II). Metastasis to the right-side cervical lymph node was subsequently detected, and a right-side radical neck dissection was performed in February 1999. Adjuvant chemotherapy with carboplatin was administered post-operatively. The patient had a history of sigmoid colon cancer and resection of a metastatic tumor in the left lung. His BMI was 23.3 kg/m^2^, and he smoked 20 cigarettes per day and drank approximately 360 mL alcohol per day. The clinical test findings included total cholesterol of 245 mg/dL and triglycerides of 508 mg/dL. On his oldest head and neck contrast-enhanced CT image stored in our hospital’s picture archiving and communication system (February 2003), the NASCET ratio at the origin of the right-side ICA was 42 % (Fig. 2a). A later head and neck contrast-enhanced CT image taken as part of his post-operative follow-up (June 2010) revealed the ratio was 70 %, indicating progression of the stenosis (Fig. 2b). Magnetic resonance angiography was performed at the Department of Neurosurgery, and stenosis of approximately 70 % according to the NASCET method was evident at the origin of the right-side ICA (Fig. 3a). An intramural thrombosis, with a high probability of unstable plaque, was revealed by hyperintensity on T1 and T2-weighted images. The situation was explained to the patient, and right-side carotid artery stenting was performed in July 2010. The stent was placed in the right-side ICA via a transfemoral artery approach under general anesthesia (Fig. 3b). Oral rosuvastatin and cilostazol were administered post-operatively, and the patient’s course has been uneventful.

## Discussion

Three factors contribute to thrombus formation: changes in vascular wall properties; changes in blood flow; and changes in blood properties. The risk factors that result in the occurrence of these contributing factors include malignant tumor, hypertension, diabetes mellitus, hyperlipidemia, smoking, and old age [10–12]. With the aging of the Japanese population, both the age and number of OSCC patients are rising [13]. It can be speculated that the host factors are also expanding as a result. Environmental factors, for example a Westernized diet, and exposure to carcinogens, for example cigarette smoke, have also been reported as risk factors for thrombus formation [14]. Therefore, the possibility that host risk factors and environmental risk factors may act synergistically to increase the risk of thrombus formation among OSCC patients must be considered.

Recent studies have reported the occurrence of ICA stenosis as a complication of treatment in patients with head and neck cancer [1–8]. Many of these reports have involved radiotherapy [1–4]. The potential causes of stenosis as a result of desquamation of vascular endothelial cells after radiation exposure include activation of platelet agglutination, intrinsic coagulation, extrinsic coagulation factors, and endothelial proliferation [15]. At the same time, reports of ICA stenosis after surgery have mostly described cases observed during the perioperative period [3–8] and, as far as we are aware, no studies have investigated the usefulness of head and neck contrast-enhanced CT for identifying progressive stenosis of the ICA after surgery, chemotherapy, or combination therapy.

In this study, post-treatment ICA stenosis was identified on the affected side in five patients, four of whom had undergone neck dissection. Neck dissection requires procedures to be performed close to the carotid artery, resulting in direct physical stimulation of this area. Radical neck dissection also requires the performance of procedures at the external carotid artery branch and dissection and severance of the internal and external jugular veins; as such, this may be one cause of changes to the blood flow [8].

According to Sabeti et al. [16], patients with progressive carotid artery stenosis have a 2.42-fold higher risk of a peripheral vascular event, for example arteriosclerosis obliterans, and a 2.0-fold higher risk of stroke.

Therefore, the results of this study suggest that, given the possibility of post-treatment vascular events, attention must be paid to subsequent changes in the ICA over time, and indicate the usefulness of head and neck contrast-enhanced CT in identifying such problems.
